# Prion Protein Does Not Confer Resistance to Hippocampus-Derived Zpl Cells against the Toxic Effects of Cu^2+^, Mn^2+^, Zn^2+^ and Co^2+^ Not Supporting a General Protective Role for PrP in Transition Metal Induced Toxicity

**DOI:** 10.1371/journal.pone.0139219

**Published:** 2015-10-01

**Authors:** Pradeep Kumar Reddy Cingaram, Antal Nyeste, Divya Teja Dondapati, Elfrieda Fodor, Ervin Welker

**Affiliations:** 1 Institute of Biochemistry, Biological Research Centre, Hungarian Academy of Sciences, Szeged, Hungary; 2 Research Centre for Natural Sciences, Hungarian Academy of Sciences, Budapest, Hungary; University of Maryland School of Medicine, UNITED STATES

## Abstract

The interactions of transition metals with the prion protein (PrP) are well-documented and characterized, however, there is no consensus on their role in either the physiology of PrP or PrP-related neurodegenerative disorders. PrP has been reported to protect cells from the toxic stimuli of metals. By employing a cell viability assay, we examined the effects of various concentrations of Cu^2+^, Zn^2+^, Mn^2+^, and Co^2+^ on Zpl (*Prnp* ^-/-^) and ZW (*Prnp* ^+/+^) hippocampus-derived mouse neuronal cells. *Prnp* ^-/-^ Zpl cells were more sensitive to all four metals than PrP-expressing Zw cells. However, when we introduced PrP or only the empty vector into Zpl cells, we could not discern any protective effect associated with the presence of PrP. This observation was further corroborated when assessing the toxic effect of metals by propidium-iodide staining and fluorescence activated cell sorting analysis. Thus, our results on this mouse cell culture model do not seem to support a strong protective role for PrP against transition metal toxicity and also emphasize the necessity of extreme care when comparing cells derived from PrP knock-out and wild type mice.

## Introduction

Transmissible spongiform encephalopathies (TSEs) or prion diseases are a family of rare and fatal neurodegenerative disorders that affect humans and many other mammals. They include Creutzfeldt-Jakob disease, Gerstmann-Sträussler-Scheinker syndrome, fatal familial insomnia and kuru in humans, bovine spongiform encephalopathy in cattle, and scrapie in sheep [1–3]. These diseases are some of the most typical representatives of conformational diseases, which also include for example Alzheimer’s disease, Parkinson’s diseases, type 2 diabetes, amyloidosis, several forms of triplet repeat diseases, and dementia with Lewy bodies [4,5]. TSEs are characterized by extensive neurodegeneration and exhibit pathological features such as neuronal loss, astrocytic activation (gliosis), and spongiform morphology in addition to clinical symptoms like cognitive and motor dysfunction [3].

Although the molecular pathogenesis of TSEs is still not well understood, it is widely accepted that the conformational transition of the native and predominantly α-helical cellular prion protein (PrP^C^) to a β-sheet rich pathogenic scrapie isoform (PrP^Sc^) that results in the accumulation of PrP^Sc^ aggregates is the central step/event of the diseases. Interestingly, the cellular isoform, PrP^C^ is also implicated in other conformational diseases where it might serve as a cell surface receptor for protein aggregates most importantly for β-amyloid oligomers [6].

The cellular form of the prion protein is expressed ubiquitously, appearing predominantly in the central nervous system (CNS) including in both neuronal and glial cells [7,8]. Following cleavage of the 22- amino acid signal peptide, the mature protein is exported to the cell surface as an N-glycosylated, glycosylphosphatidylinositol (GPI)-anchored protein [9]. Structural studies have revealed that PrP consists of a long, disordered, flexible NH_2_-proximal region and a globular COOH-proximal domain. The structurally less defined N-terminal region contains a unique, highly conserved octapeptide repeat region (OR). The OR consists of multiple copies of the eight residue sequence PHGGGWGQ [9,10], surrounded by two positively charged clusters, CC1 (aa 23–27) and CC2 (aa 95–110). The unstructured and the globular domains are linked together by a hydrophobic core (HC) region (aa 111–134) that is considered as a key region in both physiological and disease related processes involving the prion protein [3].

The exact cellular functions of PrP^C^ are still unknown. However, in the last several years various biological functions have been suggested for this protein, including signal transduction, neurotransmitter metabolism, cell adhesion, antioxidant activity, neurogenesis, immune cell activation, copper metabolism and homeostasis of trace elements [11–17]. The possible involvement of metals do not seem to be restricted only to the normal physiology of the prion protein: the direct or indirect interactions of PrP with various transition metals have been implicated in both TSE and Alzheimer's as being a contributing factor to triggering neurodegenerative condition [18–20]. Both *in vivo* and *in vitro* studies demonstrated that PrP^C^ binds divalent cations [21]. Experimental and molecular dynamics studies on recombinant PrP and PrP-derived peptides indicated the existence of a number of potential binding sites for divalent metal ions. The mostly encountered site is the OR of PrP^C^, which can bind copper, zinc, nickel, iron and manganese; among which copper shows the highest binding affinity to the OR region [22–26]. The structure and stability of the formed complexes are highly dependent on pH and metal/ligand ratio [27–29]. In the presence of sub-stoichiometric metal concentrations or acidic pH, the imidazole nitrogen atoms are the only truly effective donor atoms, for both copper and zinc. Macro-chelates are formed, in which up to four histidines bind a single metal ion. Two additional copper coordinating sites have also been identified at His-96 and His-111 in human PrP^C^. At neutral or basic pH and in the presence of concentrations of copper at least equimolar with respect to the peptide, all histidines can behave as independent coordination sites and PrP^C^ can bind up to six Cu^2+^ ions, in vivo [11] as reviewed recently [30]. In this case, the amide nitrogen atoms come predominantly from the neighboring Gly-s. Zn^2+^ is not able to displace amide protons and forms less stable complex in respect to Cu^2+^.

Although PrP^C^ has an apparent affinity toward several transition metals it is much less clear that which of these interactions is attributable to a physiological activity of PrP^C^. This has prompted a number of *in vitro* and *in vivo* studies to investigate this relation [18,31,32].

Transition metal-PrP^C^ interactions might have an impact on PrP^C^ biology by the internalization and shedding of PrP^C^ that were reported to occur as a response to transition metal stimuli [33–35]. Metals are also reported to affect PrP^C^ folding and structure and the occupancy of metal binding sites of PrP^C^ by either copper or manganese is thought to influence its conformational transition to PrP^Sc^ [36,37].

These metals are essential cofactors and are involved in a great number of critical biological processes. PrP^C^ is also proposed to affect the homeostasis of divalent cations such as copper, zinc, manganese and iron [18]. Several studies suggested that PrP^C^ is directly involved in the uptake/transport of metals, primarily copper, zinc or iron, although a direct evidence that PrP^C^ does, in fact, transport these metals is still lacking.

Free transition metal ions are especially highly effective in generating reactive oxygen species (ROS) that can induce lipid peroxidation and protein oxidation, leading to cellular damage [38,39]. Many reports showed a protective role of PrP^C^ against cellular stresses, especially, against oxidative damage, which is perhaps one of the most widely accepted functions of PrP^C^ [11,16,40–44]. Remarkably, the loss of antioxidant defense was suggested to play a major role in scrapie-infected cells [45] and prion diseases [46–49]. Regarding the mechanisms of these protective effects of PrP^C^, it was shown that cultured cells derived from *Prnp* ^−/−^ mice were more sensitive to oxidative damage and exhibited reduced superoxide dismutase (SOD) activity, when compared with WT [16]. Furthermore, recombinant PrP^C^ refolded in the presence of Cu^2+^ was reported to have SOD activity [50] although other authors found neither decreased SOD activity in *Prnp* ^−/−^ mice [32] nor SOD activity with recombinant PrP^C^ [51,52]. In addition, experiments using genetically modified mice, as well as crosses between PrP^C^ overexpressing and SOD-deficient mice, argue against such a role for PrP^C^ in vivo [53].

Alternatively, it is possible that the binding of Cu^2+^ or Zn^2+^ to PrP^C^ that induces its endocytosis is a signal for triggering antioxidative defense [16,54,55]; even though non-oxidative mechanisms are also considered [56]. Nevertheless, whereas the mechanism is not clear, a protective effect for PrP^C^ or its fragments in metal-induced toxicity has been reported by a few studies in various model systems [56–58].

Zpl cells are hippocampus-derived *Prnp* ^-/-^ immortalized cells that have been shown to be more vulnerable to apoptotic cell death than PrP^C^-expressing counterparts [59]. These altered sensitivities were shown to be related to PrP expression since reintroduction of PrP into *Prnp* ^-/-^ cells restored the viability of the cells. Here we used this model system to investigate whether these cells lacking PrP are more vulnerable to metal-induced toxicities as well. We selected four metals among which Cu^2+^, Mn^2+^ and Zn^2+^ has been studied more extensively for their interaction with PrP [11,24,60,61], while Co^2+^ that has been shown to also bind PrP [26,62] has not been studied in such details. We found no study for investigating the role of PrP^C^ in defending cells against Co^2+^ toxicity; by contrast, a protective role against Cu^2+^, Zn^2+^ and Mn^2+^ induced toxicities has been reported [56–58] in cell cultures; although the data are less univocal for Zn^2+^ [56,57].

## Materials and Methods

### Reagents and antibodies

All chemicals used were from Sigma-Aldrich unless stated otherwise. AlamarBlue cell viability reagent was from Life Technologies (DAL1100). Protease Inhibitor Cocktail and Phenylmethanesulfonyl fluoride (PMSF) were from Sigma-Aldrich (P2714 and P7626, respectively). PNGase F was from New England Biolabs (P0704), TurboFect Transfection Reagent was from Thermo Scientific (R0531), RC DC Protein Assay kit was from Bio-Rad (500–0121), Copper(II) sulfate pentahydrate, Zinc (II) sulfate heptahydrate and cobalt chloride (II) hexahydrate were from Molar Chemicals Kft, MnCl_2_ was from Sigma-Aldrich (M8054).

Anti-Prion Protein Monoclonal Antibody was from Spi-Bio (Clone SAF 32; A03202), Anti-Mouse IgG (Fab specific)–Peroxidase antibody and β-Actin antibody were from Sigma-Aldrich (A3682 and A5316, respectively), Alexa Fluor 568 Anti-Mouse IgG (H+L) was from Life Technologies (A11004).

All cell culture reagents were from Life Technologies/Gibco; high glucose Dulbecco's Modified Eagle's medium (DMEM) (41966), Fetal Bovine Serum (FBS) (10500), Penicillin Streptomycin (15070), GlutaMAX (35050).

### Cell culturing, transfection and generation of stable cell lines

Neuronal cell lines ZW 13–2 and Zpl 2–1, were established from the hippocampus of ICR (*Prnp* ^+/+^) and Zurich I *Prnp* ^−/−^ mice, respectively, by Kim and covorkers [63]. Zpl 2–1 cell line stably expressing mouse PrP and GFP (Zpl 2-1-PrP) or GFP and puromycin resistance gene (Zpl 2-1-vector) were produced as follows. Briefly, 1x10^5^ cells/well were seeded on a 6-well plate and transfected using TurboFect transfection reagent, according to manufacturer's protocol. The following plasmids were introduced to the cells: A Sleeping Beauty plasmid (pSB), containing two expression cassettes between transposon arms, one coding the Enhanced Green Fluorescent Protein (EGFP) driven by CMV promoter, and the other coding either wild type mouse prion protein (PrP) or a puromycin resistance gene driven by a CAG promoter. The second plasmid (SBx100) was coding for an active transposase enzyme to catalyze the linked integration of the two expression cassettes from the pSB plasmid. GFP positive cells were sorted 3 and 14 days post-transfection using a FACS Aria fluorescence-activated cell sorter. The cells were amplified and frost at about 35 post-transfection. The expression and localization of GFP and PrP were confirmed by western blotting and immunocytochemistry. All cells were maintained in DMEM supplemented with 10% FBS, 1% GlutaMAX and 1% penicillin-streptomycin at 37°C under 5% CO_2_.

### Western blot analysis

One 10 cm Petri dish of confluent cultured cells were washed twice with ice-cold phosphate buffered saline (PBS) and scraped in a 1 ml volume of PBS. Cells were pelleted by centrifugation at 500 ×g and 4°C for 5 min. The supernatant was removed, and the pelleted cells were resuspended and lysed in cold lysis buffer (50 mM Tris–HCl pH 7.4, 1 mM EDTA, 150 mM NaCl, 1% Triton X-100) supplemented with 1 mM phenylmethyl sulfonyl fluoride and protease inhibitor cocktail, and solubilization for 30 min on ice. Lysed cells were centrifuged at 15000 ×g and 4°C for 10 min to pellet and remove insoluble materials. Protein concentration of the soluble fraction was determined using RC-DC Protein Assay. For studies involving PNGase F digestion, each sample was treated with PNGase F as directed by the manufacturer. Briefly, each sample of 20 μg protein was denatured at 100°C for 10 minutes and incubated with and without of 1,500 units of PNGase F at 37°C for 2 hours. Samples were then analyzed by Western blot as follows. Equal amounts of total proteins, typically 10 μg were loaded on 12% polyacrylamide gels, separated by SDS-PAGE and transferred to methanol-activated Hybond-P polyvinylidene difluoride membrane (Millipore). The membrane was blocked by 5% nonfat dry milk in PBS containing 0.05% Tween 20 (PBST) for 1 h at room temperature followed by an overnight incubation with primary antibody against PrP (1:3000), or β-actin (1:10,000). Blots were then incubated with horseradish peroxidase conjugated secondary antibody. The reactive protein bands were visualized on X-ray films by Chemiluminescent substrate (Millipore) and were subsequently quantified by densitometry analysis of the gray-scale images of the scanned films using the ImageJ software.

### Immunocytochemistry

Immunocytochemistry was used to determine the expression of PrP in the neuronal cell lines used. Cells were plated at ~0.5x10^5^ cells/ml onto 8-well chamber microscope slides (Nunc, Lab-Tek II). After 24 h of seeding, cells were washed with PBS and were fixed with fresh 4% paraformaldehyde (PFA) for 10 minutes at room temperature. PFA was removed by washing the cells with PBS. Samples were blocked by adding blocking solution (1% BSA in PBS) for 45 min at room temperature, and were incubated with anti PrP (SAF-32) primary antibody at 1:250 dilution in blocking solution, overnight at 4°C. From each cell line one well was kept in blocking solution without applying the primary antibody, to serve as controls for estimating the background signal coming from unspecific binding of the secondary antibody. Next day cells were washed with blocking solution to remove primary antibody, followed by incubation with an anti-mouse Alexa Fluor 568 conjugated secondary antibody used at 1:300 dilution in blocking solution for 1 h at 37°C. Unbound antibodies were washed by PBS and cells were incubated in 100 ng/ml 4′,6-diamidino-2 phenylindole HCl (DAPI) for 5 min to stain DNA. DAPI was washed out by PBS and cells were imaged in PBS by using a confocal laser scanning microscope Fluoview FV1000 (Olympus Life Science Europa GmbH, Hamburg, Germany). Microscope configuration was as follows: UPLSAPO 60X (oil, NA:1.35) objective, zooming factor of either 4 or 1, sampling speed 4 μs/pixel, line averaging 2X, scanning mode sequential, unidirectional, excitation 405 nm (DAPI detection) and 543 nm (with Alexa568 red fluorescence detection). Counting of the PrP positive cells of the transformant Zpl 2-1-PrP and Zpl 2-1-vector cells from the microscopy pictures was performed using five pictures recorded with 60X objective, no zooming, which equaled to populations of at least 250 cells.

### Metal ion treatment of cells

When cells reached the desired confluency for a specific assay the media on top of the cells was supplemented with the indicated concentrations of either CuSO_4_, ZnSO_4_, CoCl_2_, or MnCl_2_. CuSO_4_ in all cases was administered as a 1:4 mol/mol ratio with glycine (Cu^2+^-Gly), which was pre-mixed fresh before the treatment. There was no any observable color change of the metal stock solution or metal supplemented media during the metal ion treatments, which would be indicative of a higher oxidation state of either cobalt or manganese. Treatments with metals were performed for 24 h duration before starting the assays.

### Cell viability assays and cell morphology

Cell viability was determined by using alamarBlue cell viability reagent and a microplate reader Fluoroskan Ascent FL Microplate Fluorometer and Luminometer (Thermo Scientific). Briefly, cells were seeded into 96-well flat-bottomed plates to reach 40% confluency by 24 h. The next day, the cells were either untreated (control) or treated with designated concentrations of Cu^2+^, Zn^2+^, Mn^2+^ or Co^2+^, respectively, for 24 h. The cell proliferation assay was performed according to the manufacturer’s instructions and fluorescence was measured by the microplate reader. At the indicated concentrations of the metal ions, morphological changes of the cells were also examined by using an Olympus CellR microscope and a 10X objective.

### Cell-death assays

Cell-death was measured by propidium iodide (PI) exclusion assay. Briefly, cells were seeded into 6-well flat-bottomed plates to reach 40% confluency by 24 h. The next day, cells were either untreated (control) or treated with designated concentrations of Cu^2+^, Zn^2+^, Mn^2+^ or Co^2+^, respectively, for 24 h. Treated and untreated cells were collected, including dead floating cells in the medium, and were washed twice in PBS before re-suspension in 2 μg/mL PI. Percentage of dead cells up-taking PI was measured by flow cytometry (BD FACSCalibur Flow Cytometer, BD Biosciences).

### Statistical analysis

Statistical analysis was performed on data originating from N = 3 to 5 independent experiments using Sigmaplot 12.5 software. The data are represented as the means ± standard deviation (S.D). For comparison between two samples, a two-tailed Student’s t-test was performed and significant differences were considered for p-values below 5% as follows; between treated (+) and untreated (-) cells (*p<0.05, **p<0.01 and ***p<0.001) and between treated Zw 13–2 cells to treated Zpl 2–1 cells or between treated Zpl 2-1-vector cells to treated Zpl 2-1-PrP cells (^+^p<0.05, ^++^p<0.01 and ^+++^p<0.001), respectively.

## Results

**Fig 1:**
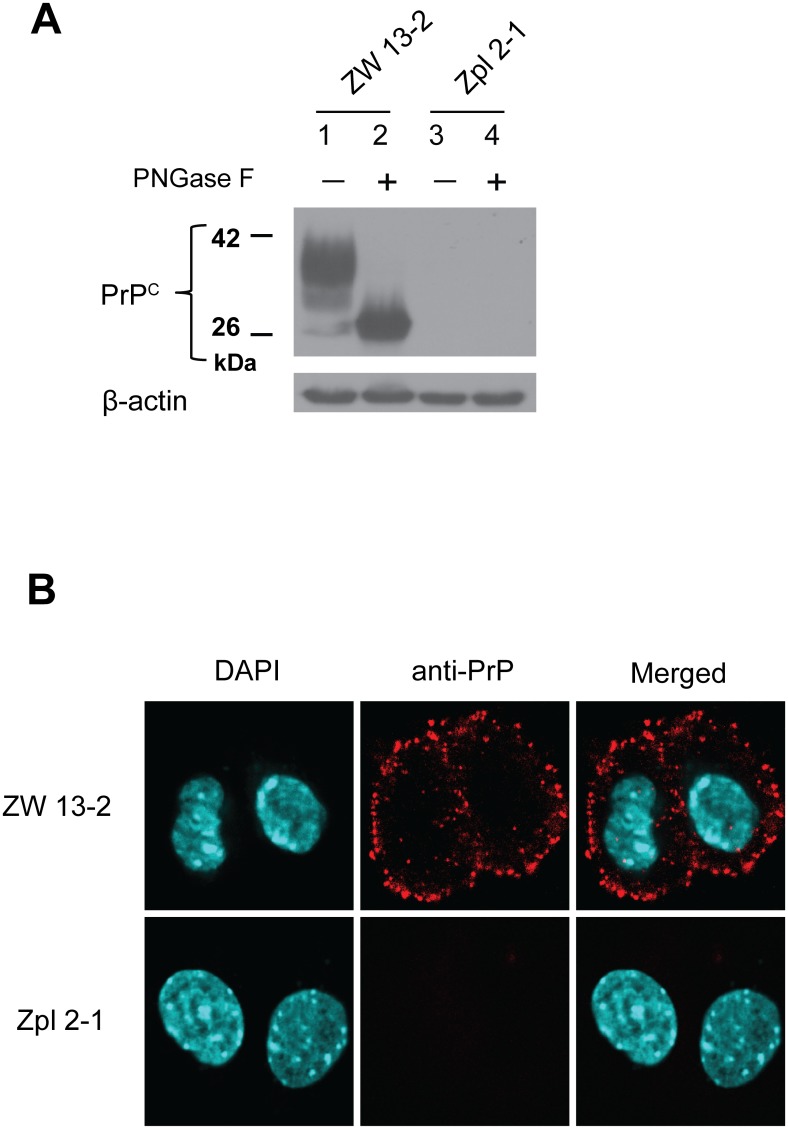
Expression levels of PrP in the ZW 13–2 and Zpl 2–1 cell lines. (A) Western blot of total cell lysates from wild type hippocampal neuronal cell line of ICR mice (ZW 13–2) and *Prnp* ^*−/−*^ hippocampal neuronal cell line of Zürich I mice (Zpl 2–1). Cell lysates were incubated either in the absence (lane 1 and lane 3) or in the presence of PNGase F (lane 2 and lane 4). Western blot analysis of PrP was carried out with monoclonal PrP antibody SAF 32. β-actin was used to confirm equal loading of proteins. (B) Immunocytochemistry verification of prion protein expression in ZW 13–2 and Zpl 2–1 cells. PrP is immunolabeled by using the monoclonal PrP antibody SAF 32 with an Alexa 568 conjugated secondary antibody (red), nucleus is stained with DAPI (cyanine blue) and merged images are shown in the last column. Pictures were recorded by using a 60X oil immersion objective and a zooming factor of 4.

The role of the prion protein in a particular cellular process, including those that are concerned with metal-PrP interrelations in *ex vivo* and *in vivo* systems is generally studied by either genetically ablating [56,63–67] or siRNA silencing [68] the expression of the prion protein. In this respect, cells that are generated from *Prnp* ^−/−^ mice are especially valuable. While some *Prnp* ^−/−^ cells are generated from mice with the ectopic expression of Doppel in the CNS [63,67], Kim and coworkers have established a series of hippocampal neuronal cell lines from Zürich I *Prnp* ^−/−^ mice along with their respective controls from the ICR *Prnp* ^+/+^ mice [63]. From this series, here we used the mouse hippocampal neuronal cell lines Zpl 2-1(*Prnp* ^−/−^) and ZW 13–2 (*Prnp* ^+/+^) that have previously been fully characterized [63]. The expression levels of the prion protein in the cell cultures maintained at our laboratory were verified before the experiments using immunoblotting and immunocytochemical analyses (Fig 1). ZW 13–2 cells have a high level of PrP^C^ expression, which is confirmed by both untreated and PNGase treated samples (lanes 1 and 2, Fig 1A). Contrary, Zpl 2–1 cells are a knockout cell line with no expression of PrP^C^; accordingly, there was no detectable band for PrP on the immunoblot (lanes 3 and 4, Fig 1A). Expression and localization of the prion protein in the two cell lines was further tested by immunocytochemical analysis (Fig 1B). The bright red immunofluorescence-staining pattern in the ZW 13–2 cells revealed that the prion protein was distributed on the surface of the cells, whereas no immunoreactivity was detected in the Zpl 2–1 cells.

**Fig 2:**
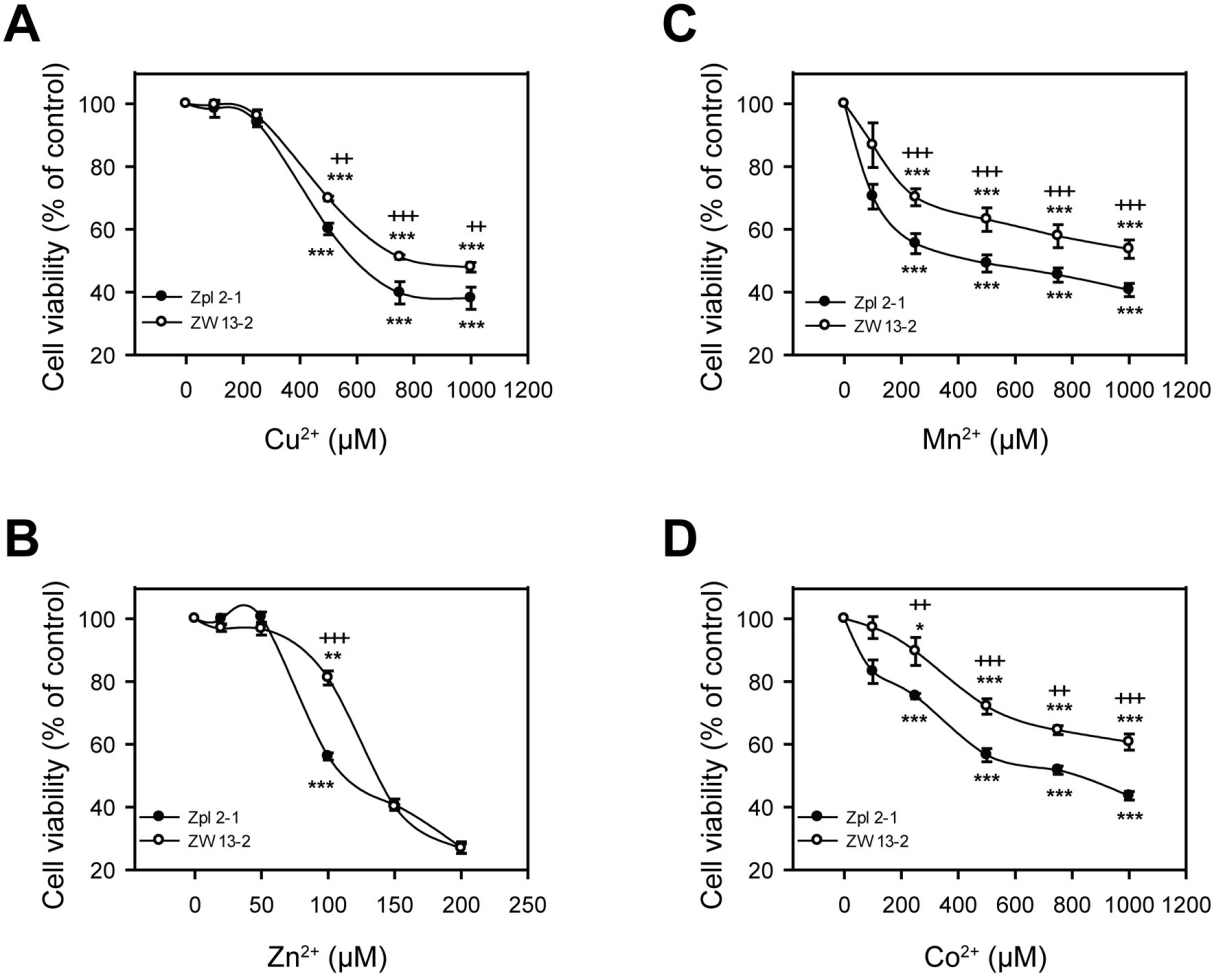
Sensitivity to transition metal-induced toxicity of ZW 13–2 and Zpl 2–1 cells. Cells were tested for survival after treatment with transition metals for 24 h, assessing cell viability by alamarBlue assay. The cell lines ZW 13–2 (open circles) and Zpl 2–1 (black circles) were treated with increasing concentrations of either Cu^2+^-Gly (A) or Zn^2+^ (B), or Mn^2+^ (C) or Co^2+^ (D). Values were compared to those of the untreated controls and are presented as percentage. The data represent the means ± standard deviation (S.D.) of minimum 3 independent experiments performed in 5 replicates. *p<0.05, **p<0.01 and ***p<0.001 indicate significant differences between treated and untreated cells; ^+^p<0.05, ^++^p<0.01 and ^+++^p<0.001 indicate significant differences between ZW 13–2 and Zpl 2–1 cells.

To test whether there is any protective role of PrP^C^ against transition metals-induced toxicity in general we aimed to assess if the two cell lines differed in susceptibility to Cu^2+^, Zn^2+^, Mn^2+^ or Co^2+^ treatments, using a range of concentrations of each metal ion and an alamarBlue-based cell viability assay. The concentracion rage where the cells proved to be sensitive to metal ion after a 24 h ttreatment was above 200 μM for copper, manganese and cobalt, and above 50 μM in the case of zinc (Fig 2). Such concentration ranges of metals at which the toxicity is observable was also found in the case of other cell lines [57,58,69]. Among the four metal ions, cells proved to be least responsive to Cu^2+^ treatment, during which cell viability started to decrease significantly only at 500 μM dose compared to untreated cells in case of both cell lines (Fig 2A). Both cell lines are most sensitive to Zn^2+^ treatment showing significant differences in the number of surviving cells compared to the untreated controls at as little as 100 μM dose (Fig 2B). Nevertheless, Zpl 2–1 cells were significantly more susceptible to Cu^2+^, Zn^2+^, Mn^2+^ and Co^2+^ toxicities than ZW 13–2 cells at all concentrations of the metal ions that fall into the toxic ranges for the cells (Fig 2).

Furthermore, we examined whether the cell lines differed in their morphological features when exposed to different doses of Cu^2+^, Zn^2+^, Mn^2+^ and Co^2+^ treatments, using light microscopy. The morphological features of ZW 13–2 and Zpl 2–1 cells exposed to each of the four metals revealed irregular shrinkage and cell rounding compared to the untreated control cells. Although the number of cells attached to the surfaces of culture dishes gradually decreased in both cell lines, there were apparently fewer attached Zpl 2–1 cells than were Zw 13–2 cells, in a dose-dependent manner for Cu^2+^ (S1A Fig), Zn^2+^ (S2A Fig), Mn^2+^ (S3A Fig) and Co^2+^ (S4A Fig) treatments. These findings clearly demonstrate that Zürich I Zpl 2–1 neuronal cells are more susceptible to Cu^2+^, Zn^2+^, Mn^2+^ and Co^2+^ induced stresses than PrP-expressing ZW 13–2 control cells. This is consistent with the contention that PrP^C^ may play a protective role against Cu^2+^, Zn^2+^, Mn^2+^ and Co^2+^ cytotoxicity.

To establish a more definite link between PrP expression and decreased sensitivity to transition metals we used the *Prnp* ^−/−^ (Zpl 2–1) cell line and stably transfected with mouse *PrP* employing a Sleeping Beauty transposase system. This system had been widely used in the last years and proved to be adequate in a wide range of studies [70,71]. The vector used also contains an EGFP expression cassette to facilitate the selection of the cells with integrated transgenes. The coupled integration of the two expression cassettes (PrP and EGFP) between the transposon arms has been demonstrated (MS in preparation) that made feasible the selection of the successful transformants by FACS. Thus, instead of cloning the stably transfected cells, a cell population could be produced with various random sites of transgene integration, averaging out the potential positional effects of individual integrations on the outcome of the experiments. An only-EGFP-expressing vector was also used for the purpose to generate control cells (Zpl 2-1-vector) along with the Zpl 2-1-PrP cells, in order to be able to rule out later the possibility of other factors than PrP^C^ expression alone, to play role in the restoration of protection against metals induced toxicity in ZW 13–2 cells.

**Fig 3:**
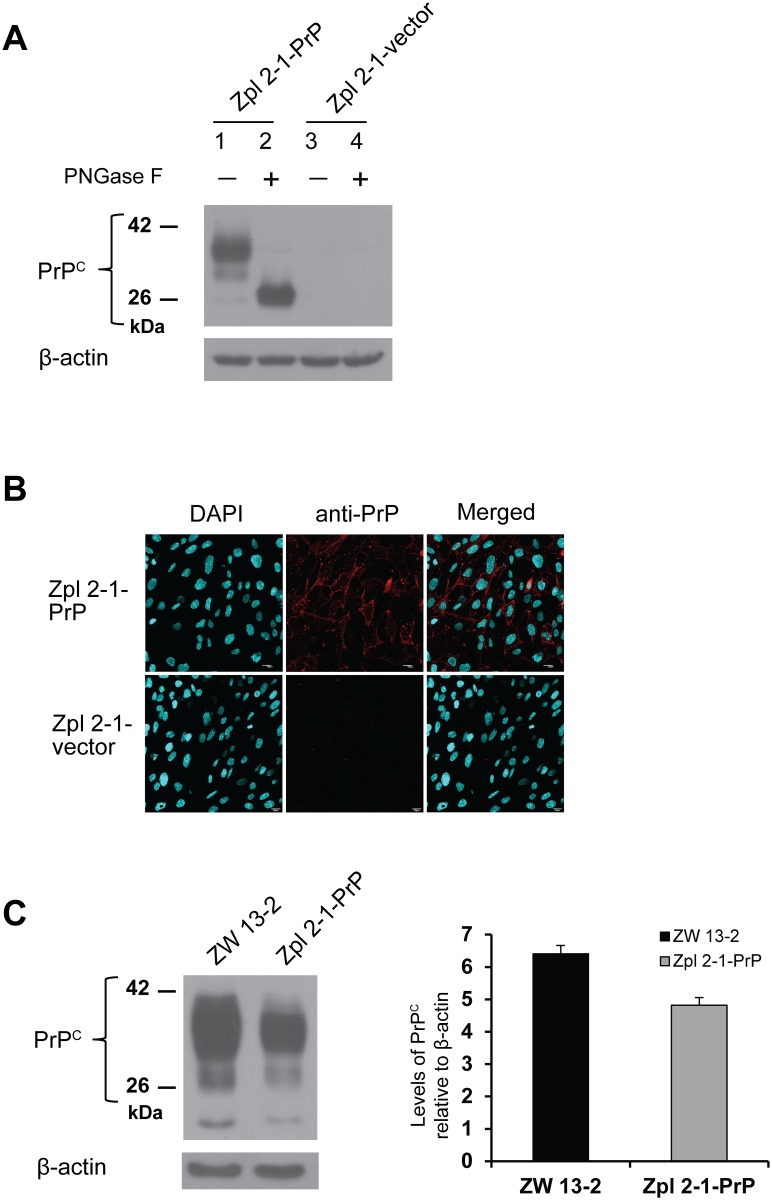
Expression levels of PrP in the generated stable cell lines. (A) Total cell lysates of stably expressing cells made from *Prnp* ^*−/−*^ hippocampal neuronal cell line of Zürich I mice (Zpl 2–1) transfected with either the empty vector (Zpl 2-1-vector) or with mouse PrP gene (Zpl 2-1-PrP). Cell lysates were incubated in the absence (lane 1 and lane 3) or presence of PNGase F (lane 2 and lane 4). Western blot analysis was carried out with monoclonal PrP antibody SAF 32. β-actin was used to confirm equal loading of proteins. (B) Immunocytochemistry performed to verify prion protein expression in Zpl 2-1- PrP and Zpl 2-1-vector cells. PrP is immunolabeled by the monoclonal PrP antibody SAF 32 and an Alexa 568 conjugated secondary antibody (red), nucleus is stained with DAPI (cyanine blue) and merged image is shown in the last column. Pictures were recorded using a 60X oil immersion objective with no zooming. (C) Relative expressions of PrP from ZW 13–2 and Zpl 2-1-PrP (left panel). The expression of PrP^C^ is quantified using densitometry analysis (right panel), the bars represent the average value obtained from three independent western blots and the error bars represent the standard deviations.

The appropriate expression level and the proper processing of the prion protein were confirmed in the established cell populations using immunoblot and immunocytochemical analyses. Total cell lysates of Zpl 2-1-vector and Zpl 2-1-PrP hippocampal neuronal cells were collected and were either left untreated or were treated by PNGase F before immunoblotting with monoclonal PrP antibody SAF 32 (Fig 3A). The Zpl 2-1-vector cells, not expected to express PrP^C^, show no detectable bands for PrP (lane 3 and 4, Fig 3A), whereas, the Zpl 2-1-PrP cells, exhibit a well-detectable level of PrP^C^ expression with proper N-glycosylation as judged by the bands in the PNGase treated and untreated samples (lanes 1 and 2, Fig 3A). Expression and correct localization of the prion protein was further confirmed by immunocytochemical analysis (Fig 3B). The bright red immunofluorescence-staining pattern in the Zpl 2-1-PrP cells revealed that the prion protein was distributed on the surface of the cells, whereas no immunoreactivity was detected in the Zpl 2-1-vector cells. The percentage of transformant cells was estimated based on the microscopy pictures using the counts of DAPI stained cells as the total cells, and the Alexa568 positive cells as counts of the transformant cells. We found that 90 (+/- 1.7) % of the Zpl 2-1-PrP cells express PrP, whereas, we found no positively stained cells of the vector-expressing cells. To compare the expression levels of PrP^C^ in ZW 13–2 and Zpl 2-1-PrP cells, western blot analysis was performed (Fig 3C, left panel). The expression of PrP^C^ was quantified using densitometry analysis of the bands with normalization to the band of β-actin as loading control (Fig 3C, right panel) and shows that PrP^C^ expression is comparable in Zpl 2-1-PrP and ZW 13–2 cells.

**Fig 4:**
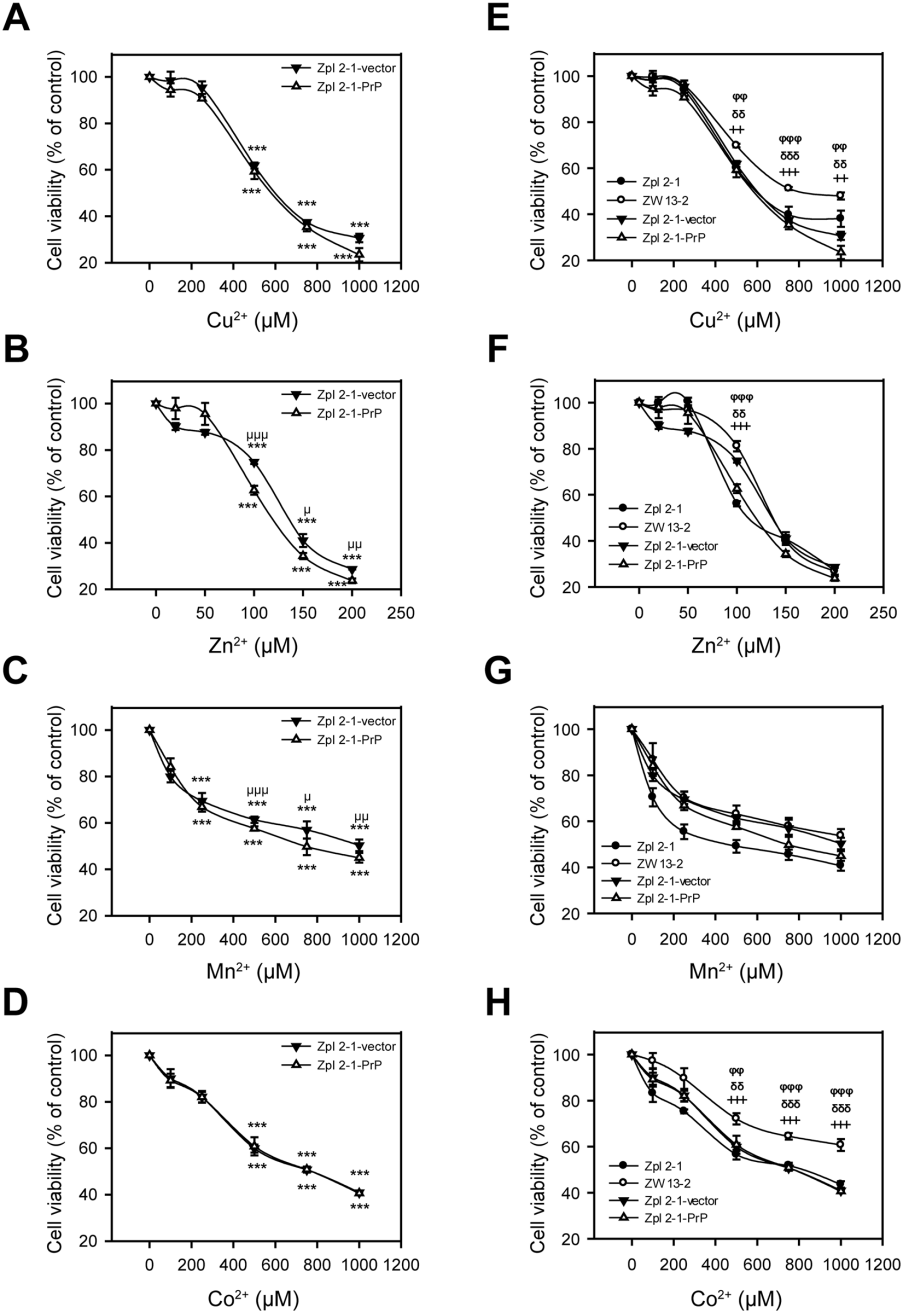
The effect of the presence of PrP^C^ on the susceptibility of cells to transition metal-induced toxicity. Cells were tested for survival after treatment with transition metals for 24 h and cell viability was determined using alamarBlue assay. Values are compared to those of the untreated controls and are expressed as percentage. The cells Zpl 2-1-PrP (open triangles) and Zpl 2-1-vector (black triangles) are compared (panels A through D) during treatments with increasing concentrations of metals, as follows: Cu^2+^-Gly (A), of Zn^2+^ (B), Mn^2+^ (C) and Co^2+^ (D). The cell lines ZW 13–2 (open circles), Zpl 2–1 (black circles) were treated similarly with increasing concentrations of metals and all four types of cells are compared on panels E through H, as follows: increasing concentration of Cu^2+^-Gly (E), of Zn^2+^ (F), Mn^2+^ (G) and of Co^2+^(H). The data are presented as means ± standard deviation (S.D.) of minimum 3 independent experiments performed in 5 replicates. *p<0.05, **p<0.01 and ***p<0.001 indicate significant differences between treated and untreated cells on panels A through D; ^μ^p<0.05, ^μμ^p<0.01 and ^μμμ^p<0.001 indicate significant differences between treated Zpl 2-1-PrP cells and treated Zpl 2-1-vector cells; ^+^p<0.05, ^++^p<0.01 and ^+++^p<0.001 indicate significant differences between the treated ZW 13–2 cells and treated Zpl 2–1 cells; ^δ^p<0.05, ^δδ^p<0.01 and ^δδδ^p<0.001indicate significant differences between the treated ZW 13–2 cells and treated Zpl 2-1-vector cells, and ^φ^p<0.05, ^φφ^p<0.01 and ^φφφ^p<0.001 indicate significant differences between the treated ZW 13–2 cells and treated Zpl 2-1-PrP cells.

To test whether the sole presence of PrP is responsible for the different susceptibilities of PrP-expressing ZW 13–2 compared to *Prnp* ^−/−^Zpl 2–1 against metal toxicity, we treated Zpl 2-1-PrP and Zpl 2-1-vector cells characterized above (Fig 3) with Cu^2+^, Zn^2+^, Mn^2+^ and Co^2+^ at the same concentrations as on Fig 2 and assessed their survival using alamarBlue-based assay (Fig 4A, 4B, 4C and 4D). Treatment with Cu^2+^, Zn^2+^, Mn^2+^ and Co^2+^ decreased significantly the cell viabilities compared to the untreated controls and at the same metal ion concentrations as in the case of ZW 13–2 and Zpl 2–1 cells. Interestingly, Zpl 2-1-PrP and Zpl 2-1-vector cells exhibit similar sensitivities, without any significant difference, in case of each metal concentration tested. These findings argue that PrP is not the sole reason for the increased resistance of ZW 13–2 cells compared to Zpl 2–1 cells. Further, when the cell survival data of all four types of cells, ZW 13–2, Zpl 2–1, Zpl 2-1-PrP and Zpl 2-1-vector are compared (Fig 4E, 4F, 4G and 4H) it can be seen that in case of Cu^+2^, Zn^+2^, and Co^+2^ the viabilities of Zpl 2-1-PrP and Zpl 2-1-vector cells are significantly lower than that of ZW 13–2 cells, whereas, no significant difference is observed in case of Mn^+2^ treatment.

Subsequently, we examined whether Zpl 2-1-PrP and Zpl 2-1-vector cells differ in their morphological features when treated with Cu^2+^, Zn^2+^, Mn^2+^ or Co^2+^ using light microscopy. The observed morphological features of cells exposed to Cu^2+^, Zn^2+^, Mn^2+^ or Co^2+^ revealed irregular shrinkage and cell rounding as compared with the untreated control cells. The number of cells attached to the surfaces of culture dishes gradually decreased in a dose-dependent manner for Cu^2+^ (S1B Fig), Zn^2+^ (S2B Fig), Mn^2+^ (S3B Fig) and Co^2+^ (S4B Fig) treatments, and in a similar manner for both types of cells.

**Fig 5:**
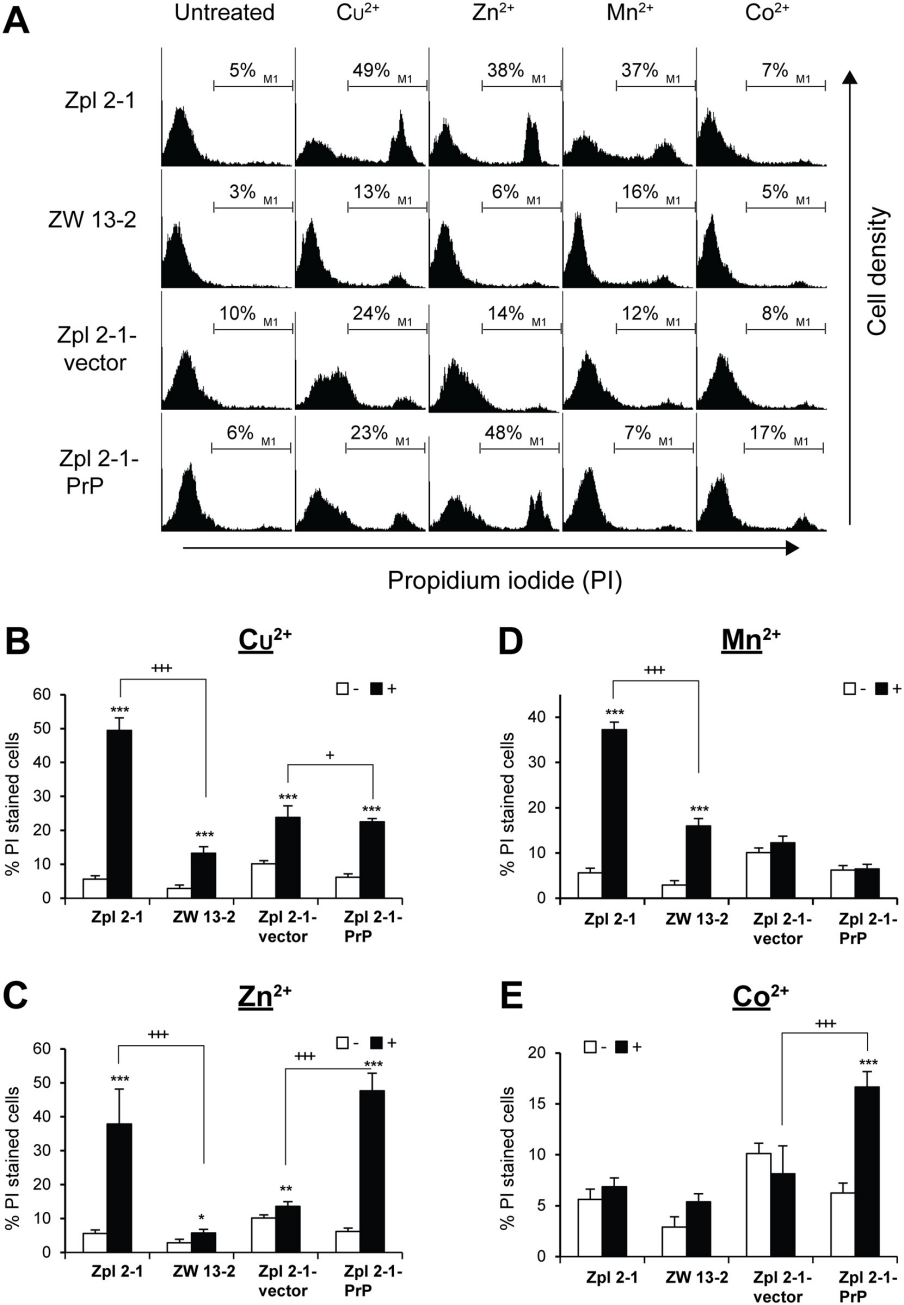
The effect of the presence of PrP^C^ on transition metals induced cell death. Cells were tested for cell death after treatment with transition metals for 24 h, cell death was measured by propidium iodide (PI) exclusion assay. (A) Zpl 2–1, ZW 13–2, Zpl 2-1-vector and Zpl 2-1-PrP cells were treated either with 750 μM of Cu^2+^-Gly (1:4 mol/mol), or with 100 μM of Zn^2+^, or 500 μM of Mn^2+^ or 750 μM of Co^2+^. Dead cells were stained with PI, and histograms were obtained by flow cytometric analysis. M1 represents the population of PI positive cells. The bar graphs on the panels b through e indicate the average of PI positive cells in case of 750 μM of Cu^2+^ treatment (B), 100 μM of Zn^2+^ treatment (C), 500 μM of Mn^2+^ treatment (D) and 750 μM of Co^2+^ treatment (E), respectively. Experiments were performed three times in duplicates, and data represent the mean ± standard deviation (S.D.). *p<0.05, **p<0.01 and ***p<0.001 indicate significant differences between treated (+) and untreated (-) cells; ^+^p<0.05, ^++^p<0.01 and ^+++^p<0.001 indicate significant differences between the ratios obtained for treated ZW 13–2 cells to treated Zpl 2–1 cells, and treated Zpl 2-1-vector cells to treated Zpl 2-1-PrP cells, respectively.

Next, we attempted to check the effect of the presence of PrP^C^ on transition metal-toxicity by assessing the cell death as a complementary approach. ZW 13–2, Zpl 2–1, Zpl 2-1-PrP and Zpl 2-1-vector cells were treated with the four transition metal ions Cu^2+^, Zn^2+^, Mn^2+^ and Co^2+^ for a period of 24 h, after which the cell death was measured by propidium iodide (PI) exclusion assay. Zpl 2–1, ZW 13–2, Zpl 2-1-vector and Zpl 2-1-PrP cells were treated either with 750 μM Cu^2+^, or with 100 μM of Zn^2+^, or 500 μM of Mn^2+^ or 750 μM of Co^2+^, respectively. Dead cells were stained with PI and the histograms obtained by flow cytometry analysis were compared (Fig 5A). Exposure to Cu^2+^, Zn^2+^, and Mn^2+^ for 24 h resulted in increase of PI positive cells in most cell lines and conditions (Fig 5B, 5C, 5D and 5E). However, the number of PI positive cells was significantly less in ZW 13–2 as compared to Zpl 2–1 lines in case of Cu^2+^, Zn^2+^ and Mn^2+^ (Fig 5B, 5C and 5D) but not in case of Co^2+^ treatments. Co^2+^ was not significantly toxic to either Zpl 2–1 or ZW 13–2 cells compared to untreated control cells (Fig 5E). In case of Zpl 2-1-vector and Zpl 2-1-PrP cells, however, such results could not be observed; the Zpl 2-1-PrP cell population had significantly more PI positive cells compared to Zpl 2-1-vector cells when treated by Cu^2+^, Zn^2+^ and Co^2+^ (Fig 5B, 5C and 5E), with both lines being unresponsive to manganese treatment. These data indicate that PrP^C^-expressing Zpl 2-1-PrP cells did not gain more resistance to any of the metals tested and at the concentrations applied, compared to PrP-lacking Zpl 2-1-vector cells.

## Discussion

A protective role against toxicity conferred by transition metal ions has been suggested for the prion protein in cell culture model systems [56–58]. Here we explored a *Prnp* ^-/-^ neuronal cell line (Zpl 2–1) of hippocampal origin as model system, along with its PrP expressing control ZW 13–2 cells, to test whether PrP has any general protective effect against transition metal toxicity; testing Cu^2+^, Zn^2+^, Mn^2+^ and Co^2+^ toxicities. Zpl 2–1 has been shown to be more vulnerable to serum deprivation and oxidative damage induced by H_2_O_2_ than its PrP-expressing counterpart [59,72,73]. Reintroduction of PrP restored the viability of Zpl cells, thus, it seems to be a relevant model for assessing the effect of PrP^C^ on metal-induced toxicity as well.

Metals might exert their toxic effects via multiple routes and PrP^C^ has been reported to interfere with a handful of pathways leading to cell death [54,74–77]. Thus, instead of dissecting a specific pathway to monitor the effect of PrP^C^ we preferred to choose methods that report rather on the *overall* viability of the cells, such as the alamarBlue viability assay and a propidium iodide-based dye exclusion assay, and examined the overall morphological changes induced by the application of various concentrations of the four transition metals.

Since alamarBlue may be reduced by reductases located in either the cytoplasm or the mitochondria its reduction may signify an impairment of the cellular metabolism and is not necessarily specific to interruption of electron transport and mitochondrial dysfunction [78]. By contrast, propidium iodide reports on the intactness of the plasma membrane.

PrP-expressing ZW cells proved to be more resistant against toxicities of all four metals examined when compared to PrP-ablated Zpl cells. These results seem to support a protective role for PrP against transition metal toxicity involving not only Cu^2+^ and Mn^2+^ but Zn^2+^ that is a rather controversial issue and Co^2+^ for which such a role has not been demonstrated [56,57]. However, when we attempted to establish a more definite link between the expression of PrP and the decreased sensitivity to transition metals we found quite surprising results.

All three approaches invariably failed to reveal any significantly increased resistance due to the presence of PrP^C^, to any of the metal-induced toxicities examined, when PrP^C^ was overexpressed in Zpl cells. Thus, we could not establish a clear link between the prion protein expression and a decreased sensitivity to metal toxicity. These are interesting results, since using other model systems and approaches, a protective role has been assigned to PrP^C^ in case of copper [56], manganese [58] or zinc [57].

Most of the studies concerning PrP^C^-copper interactions focused on the actual binding event taking place *in vitro*, monitored the internalization of PrP^C^ upon exposure to Cu^2+^ as a direct indication of *in vivo* PrP^C^-Cu^2+^ interaction and/or run assays to show the impact of the ablation of PrP^C^ expression on steps of the oxidative stress response [11,33,56]. Only one study aimed to examine if PrP^C^ expression confers resistance against copper toxicity. Haigh and Brown applied Cu^2+^ alone or chelated with glycine to PrP-expressing and control F14 cells and reported that PrP^C^ decreased the vulnerability of the cells to copper treatment. They reported that copper when chelated with glycine generates no ROS and does not trigger oxidative cell response. Nevertheless, a protection against these "nonoxidative components" of copper toxicity is also provided by PrP^C^ [56]. In case of Mn^2+^ that also forms chelate with glycine, although a weaker one, they found no difference between the toxic effects of the treatments with or without glycine. Nevertheless, PrP^C^ expression made F14 cells less vulnerable to Mn^2+^ toxicity at the higher concentrations of the manganese applied [56]. Interestingly, the same F14 cell lines, (with or without PrP^C^ expression), in the same study exhibited no different viability when incubated with various concentration of Zn^2+^ [57]. Choi et al. corroborated the former results of Haigh and Brown on manganese examining CF10 cell lines with or without PrP^C^ expression. Mn^2+^ treatment generated more ROS, caused more pronounced depletion of GSH and higher caspase 3 and 9 activation in the PrP^C^ ablated CF10 cell line [58]. Interestingly, they concluded that PrP^C^ likely interferes with the uptake of Mn^2+^ (in addition to its effect on oxidative cell response) [58]. Regarding Zn^2+^, in contrast to the results of Haigh and Brown [56], Rachidi et al. had shown that A74 cells that are devoid of PrP expression became more resistant to Zn^2+^ toxicity when PrP-expression was induced [57]. They suggested that PrP^C^ mediates intracellular redistribution of the interchangeable Zn^2+^ rather than interfering with cellular Zn^2+^ uptake. Furthermore, they put forward an explanation how PrP attenuates metal induced oxidative stress response: PrP-expression upregulates thionein expression that sequesters metals thereby decreasing their ROS generating capacity.

It is not easy to compile all the existing data into a coherent interpretation. The cell lines used in these studies had been derived from different tissues and species. A74 cells are from rabbit kidney while the mouse neuronal cells are from cerebellar (F14) or hippocampal (Zpl) origins. Thus, these results might reflect the various roles PrP plays in defending the cells against toxicity induced by the four transition metals investigated.

It is also clear that metal induced toxicity involves complex and divergent pathways where subtle differences that are not apparently related to the transgene-expression, alter significantly the sensitivity of the cells. For example, Zpl cells become more resistant to Mn^2+^ treatment by the introduction of the vector control expressing only a GFP protein (Fig 4G and Fig 5D). Haigh and Brown found similar outcomes: F14 cells became a magnitude more resistant to Cu^2+^ treatment by the introduction of a control vector expressing only a GPI-anchored GFP, as apparent if one compares Fig 3*a* and *b* in their results [56]. What they found is a greater difference than the one they observed as a consequence of PrP expression between the viability of the GFP-GPI and GFP-PrP cell lines (Fig 3a and 3b in [56]) during cooper treatment. In the study of Haigh and Brown [56] the stable cell lines were created by random integration of the transgene using a common transfection protocol of the plasmids (Fugene 6, Roche, UK), whereas in our case the sleeping beauty transposase method was used. This indicates that this effect is probably independent of the method used for insertion of the transgene. Thus, it is also possible that the effect of PrP expression on these transition metal toxicities is just not robust enough to be discernible in all cases.

One concern might be the use of the CAG promoter instead of the natural PrP promoter in these studies. Fig 3 shows that the expression level in Zpl-PrP is quite moderate and comparable to that of the ZW cells. However, the regulation of the PrP promoter is not displayed by this construct. Out of the four metals examined in this study, copper is reported to enhance transcription of the prion protein. However, zinc does not enhance transcription from the prnp gene. Thus, this is not a likely mechanism for providing a general protection against transition metals. In another studies, Haigh and Brown used CMV promoter while Rachidi et al employed an inducible TRE promoter, none of them is able to provide a regulation similar to that of the natural PrP promoter, yet, found a protective effect of PrP against copper, manganese and zinc toxicity, respectively. This further supports that the artificial promoter used is not the reason of the lack of PrP’s protective effect in our experiments

An additional factor to be taken into account might be the difference in the methods used. For F14 and A74 cells the viability was monitored with MTT assay, while CF10 cells were analyzed by the Trypan blue exclusion method. Zpl cells were assayed in our study by both approaches, using an alamarBlue viability test and a propodium iodide exclusion method. Interestingly, even when we compared the same cells (Zpl 2-1-PrP and ZW 13–2) for the same metal (Mn^2+^) the relative sensitivities of the cells varied with the two methods used. Zpl 2-1-PrP cells were found more sensitive when assayed for cell viability (alamarBlue) and less sensitive when assayed by dye-exclusion (propidium-iodide) than ZW 13–2 cells (Fig 4G and Fig 5D). Also, if one compares the vector cells to the PrP-expressing cells, treatments with three metals (copper, zinc and cobalt) resulted in altering sensitivities when examined with the two methods. The vector cells treated by cobalt are significantly more resistant than the PrP-expressing cells when examined by propidium iodide while there is no significant difference between them when the cell viability assay is performed. By contrast, in case of the manganese treatment they prove to be more resistant than PrP-expressing one when assessed by viability assay, but there is no significant difference between the cell lines when probed by the propidium iodide method. In contrary, against copper the PrP-expressing cells are marginally significantly more resistant compared to the vector control cells when assayed by propidium iodide while no difference is detected in the cell viability assay. Interestingly, only in case of the zinc treatment and only the vector expressing cells show resistance by both techniques, with propidium iodide and with alamarBlue assays, at 100 μM concentration at which the propidium iodide experiments were conducted (Fig 5C). From these observations, two points may be made: the relative sensitivities of the four cells on Fig 4 are varied among the four metals, which in turn suggests no identical mechanism of actions nor common way of interference for PrP. These results also show that the methods used might be influential, and these might contribute to the differences seen in respect to the role of PrP in transition metal toxicities of F14, CF10, A74 and Zpl cells [56,58,63,79].

It might also be worth to note that Haigh and Brown used a GFP-PrP fusion protein. We found that a fluorescent protein fusion tag interferes with the activity of a mutant PrP; specifically, a ΔCR PrP (Delta105-125) confers drug hypersensitivity to the cells in the absence of WT-PrP [80], whereas, fusion of the fluorescent protein to ΔCR PrP abolishes this effect (unpublished result). Contrary, N-terminally fused GFP-PrP responds to Cu^2+^ stimuli with internalization similarly to the WT protein [77]. Thus, it is likely that PrP-metal interactions are not disturbed by the N-terminal GFP tag, however, it might influence the interactions of PrP with other cell constituents.

Another critical issue is the possibility of a site specific effect of the integration of the transgene and the possible clonal variability. Here we used a sleeping beauty transposase for the effective insertion of the transgenes. Sleeping beauty inserts DNA between its transposon arms to the genome with little sequence preference. Its efficiency is higher than that of the random integration by up to two orders of magnitude. In our study, using sleeping beauty resulted cell populations with a few thousands of independent cells with unique integration sites. Thus, considering the size of the human genome, the likeliness that any gene involved in PrP associated resistance against metal toxicity is invalidated by the transgene integrations is very low. Conceivably, such subpopulation is much smaller than 1%. In addition, Fig 3C shows that close to 100% of Zpl-PrP cells express PrP, suggesting that only very few positions of the thousands of integrations interfere with the transgene expression ruling out the possibility that a non-expressing sub population muddies the cumulative protective involvement of PrP in the cellular response to divalent cations. By contrast, F14/GFP-GPI, F14/GFP-PrP, CF10/PrP^C^, CF10/PrP^KO^ and A74 cell lines were derived from a few unique integration sites. Without examining cell lines originating from different clones it is difficult to rule out a possible effect of the integration sites on the outcome of the experiments. Nevertheless, it is apparent from this short review of the available data that there is no single technical factor that could explain appropriately these seemingly incoherent results. It is likely, that if PrP^C^ has any measurable impact on the complex and divergent pathways of metal toxicity, it is not a robust, general effect that is easily discernible in all types of cells.

## Conclusions

Although Zw cells established from WT mice are more resistant to all four metal ions tested, we could not establish a clear link between prion protein expression and an increased resistance to metal toxicity, since PrP expression does not confer increased resistance to Zpl cells as compared to GFP expression alone. Thus, the increased resistance of Zw cells are either not related to PrP^C^ expression, or at least PrP^C^ expression is not a sole factor necessary for the increased resistance.

## Supporting Information

S1 Fig Morphological appearance of ZW 13–2, Zpl 2–1, Zpl 2-1-vector and Zpl 2-1-PrP. ZW 13–2 and Zpl 2–1 (A) and of Zpl 2-1-vector and Zpl 2-1-PrP (B) cells, when treated with the indicated concentrations of Cu^2+^-Gly. Transmission light microscopy images of cells recorded using a 10X objective.
(TIF)

S2 Fig Morphological appearance of ZW 13–2, Zpl 2–1, Zpl 2-1-vector and Zpl 2-1-PrP. ZW 13–2 and Zpl 2–1 (A) and of Zpl 2-1-vector and Zpl 2-1-PrP (B) cells treated with the indicated concentrations of Zn^2+^. Transmission light microscopy images of cells, acquired using a 10X objective.
(TIF)

S3 Fig Morphological appearance of ZW 13–2, Zpl 2–1, Zpl 2-1-vector and Zpl 2-1-PrP. ZW 13–2 and Zpl 2–1 (A) and of Zpl 2-1-vector and Zpl 2-1-PrP (B) cells treated with the indicated concentrations of Mn^2+^. Transmission light microscopy images of cells acquired using a 10X objective.
(TIF)

S4 Fig Morphological appearance of ZW 13–2, Zpl 2–1, Zpl 2-1-vector and Zpl 2-1-PrP. ZW 13–2 and Zpl 2–1 (A) and of Zpl 2-1-vector and Zpl 2-1-PrP (B) cells treated with the indicated concentrations of Co^2+^. Transmission light microscopy images of cells recorded using a 10X objective.
(TIF)
