# Supplementary figures and images for: Prion Protein Does Not Confer Resistance to Hippocampus-Derived Zpl Cells against the Toxic Effects of Cu2+, Mn2+, Zn2+ and Co2+ Not Supporting a General Protective Role for PrP in Transition Metal Induced Toxicity
Source: PLoS One. 2015 Oct 1;10(10):e0139219. doi: 10.1371/journal.pone.0139219 (PMC4591282; doi:10.1371/journal.pone.0139219)

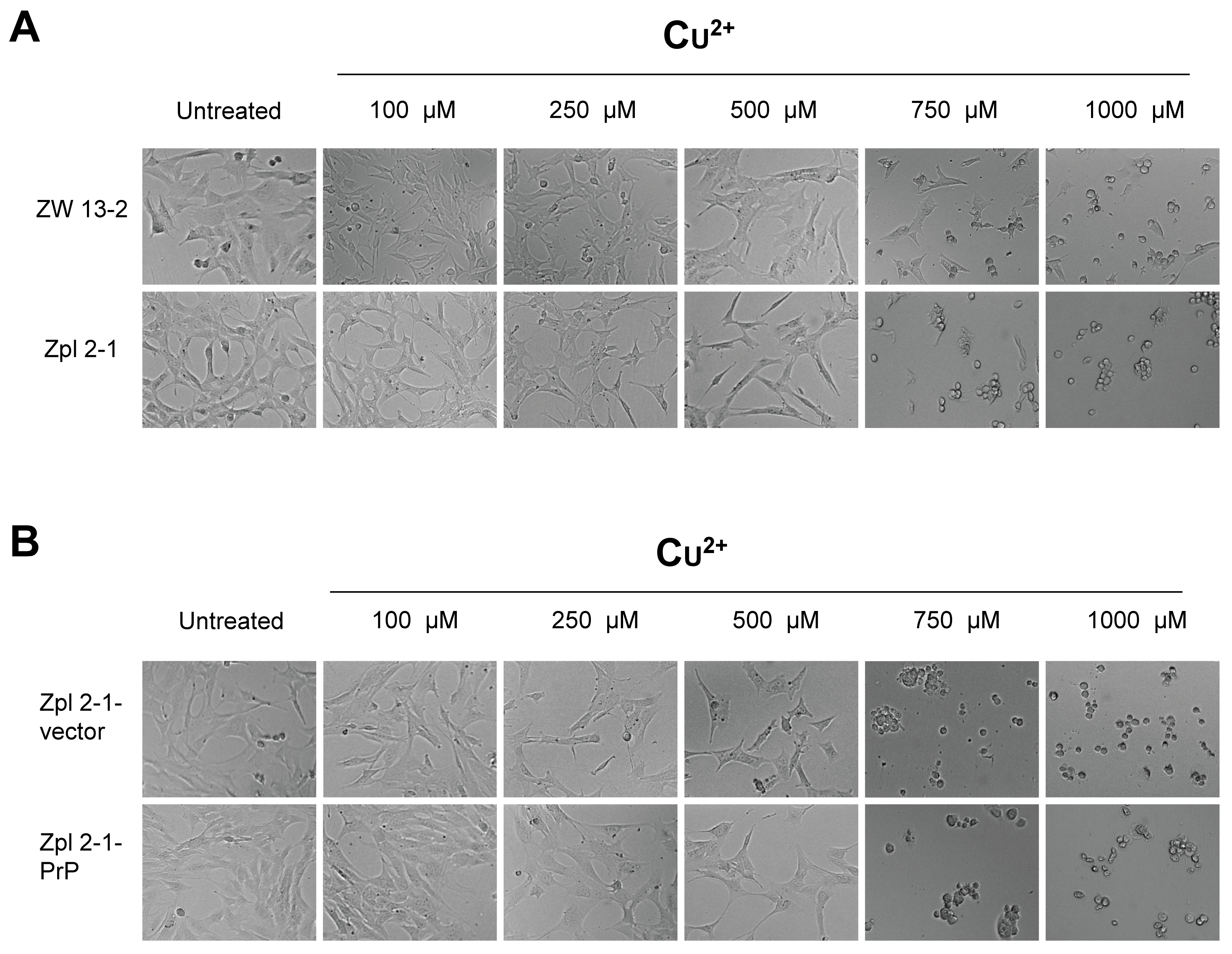

Supplement: S1 Fig — ZW 13–2 and Zpl 2–1 (A) and of Zpl 2-1-vector and Zpl 2-1-PrP (B) cells, when treated with the indicated concentrations of Cu2+-Gly. Transmission light microscopy images of cells recorded using a 10X objective. (TIF) [file pone.0139219.s001.tif]

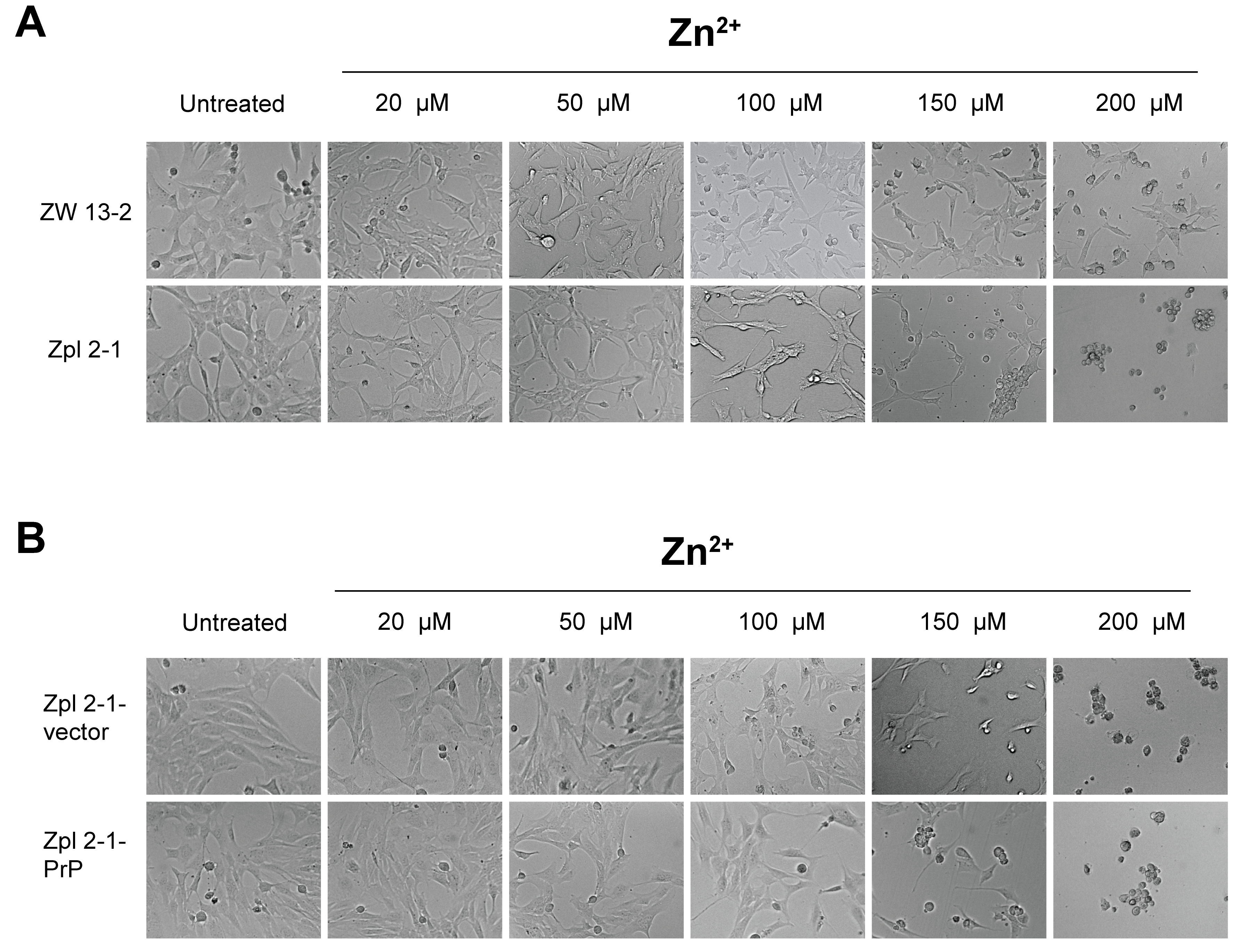

Supplement: S2 Fig — ZW 13–2 and Zpl 2–1 (A) and of Zpl 2-1-vector and Zpl 2-1-PrP (B) cells treated with the indicated concentrations of Zn2+. Transmission light microscopy images of cells, acquired using a 10X objective. (TIF) [file pone.0139219.s002.tif]

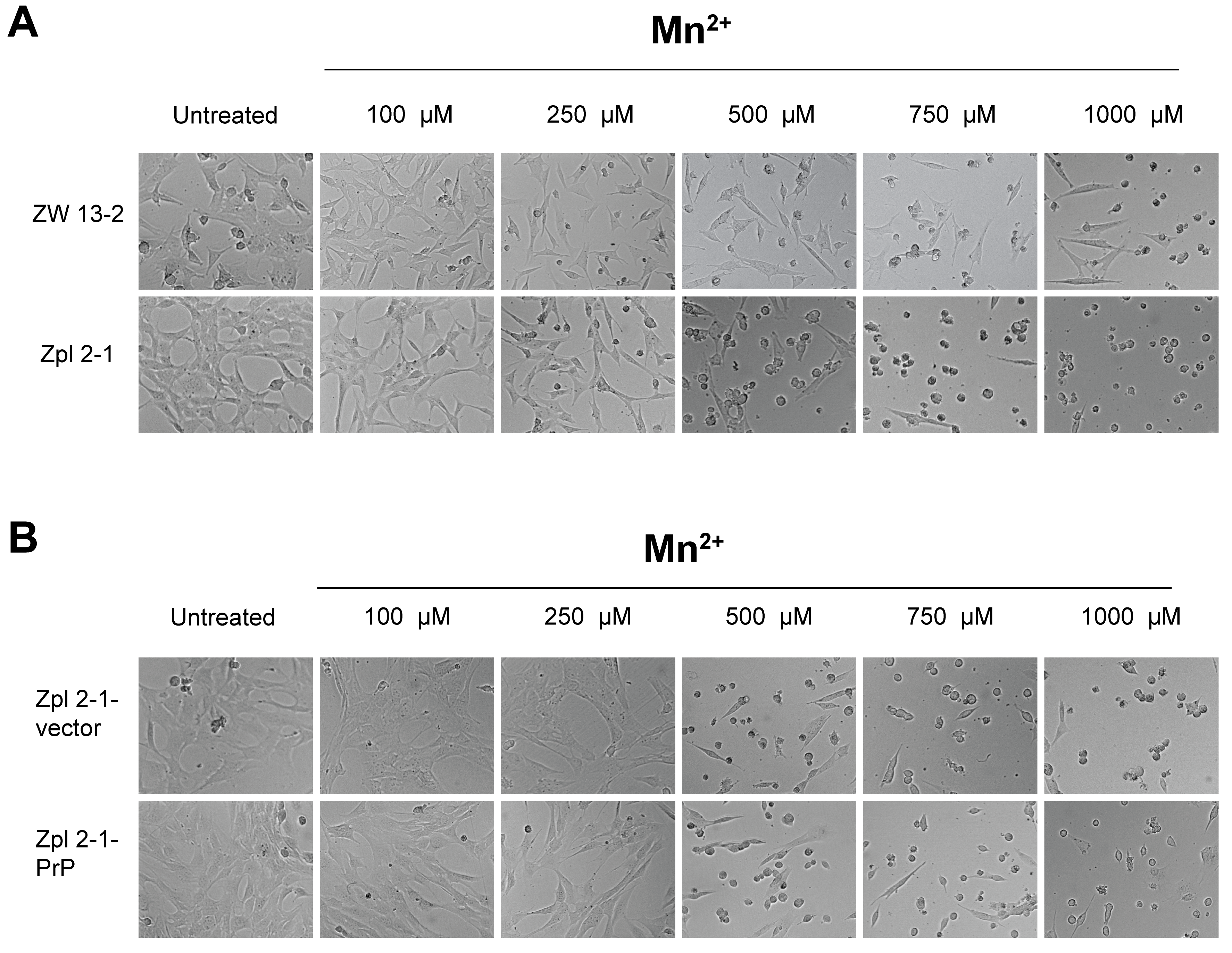

Supplement: S3 Fig — ZW 13–2 and Zpl 2–1 (A) and of Zpl 2-1-vector and Zpl 2-1-PrP (B) cells treated with the indicated concentrations of Mn2+. Transmission light microscopy images of cells acquired using a 10X objective. (TIF) [file pone.0139219.s003.tif]

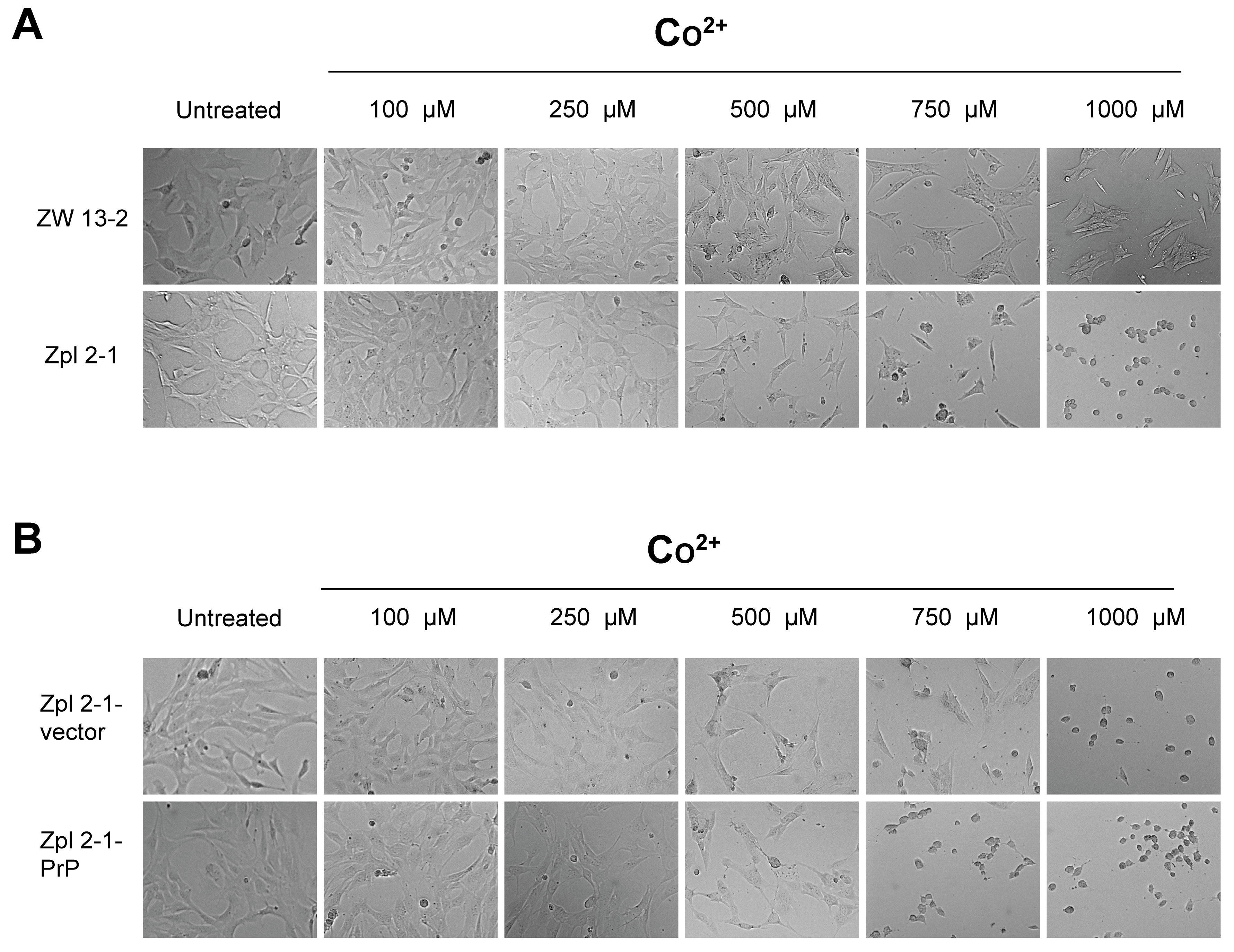

Supplement: S4 Fig — ZW 13–2 and Zpl 2–1 (A) and of Zpl 2-1-vector and Zpl 2-1-PrP (B) cells treated with the indicated concentrations of Co2+. Transmission light microscopy images of cells recorded using a 10X objective. (TIF) [file pone.0139219.s004.tif]
